# The CloudUPDRS smartphone software in Parkinson’s study: cross-validation against blinded human raters

**DOI:** 10.1038/s41531-020-00135-w

**Published:** 2020-12-08

**Authors:** Ashwani Jha, Elisa Menozzi, Rebecca Oyekan, Anna Latorre, Eoin Mulroy, Sebastian R. Schreglmann, Cosmin Stamate, Ioannis Daskalopoulos, Stefan Kueppers, Marco Luchini, John C. Rothwell, George Roussos, Kailash P. Bhatia

**Affiliations:** 1 Department of Clinical and Movement Neurosciences, UCL Queen Square Institute of Neurology, London, UK; 2 Department of Biomedical, Metabolic and Neural Sciences, University of Modena and Reggio Emilia, Modena, Italy; 3 Queen Square Movement Disorders Centre, Department of Clinical and Movement Neurosciences, UCL Queen Square Institute of Neurology, London, UK; 4 Birkbeck College, University of London, London, UK; 5 Benchmark Performance Ltd, Colchester, UK

**Keywords:** Parkinson's disease, Predictive markers

## Abstract

Digital assessments of motor severity could improve the sensitivity of clinical trials and personalise treatment in Parkinson’s disease (PD) but have yet to be widely adopted. Their ability to capture *individual* change across the heterogeneous motor presentations typical of PD remains inadequately tested against current clinical reference standards. We conducted a prospective, dual-site, crossover-randomised study to determine the ability of a 16-item smartphone-based assessment (the index test) to predict subitems from the Movement Disorder Society-Unified Parkinson’s Disease Rating Scale part III (MDS-UPDRS III) as assessed by three blinded clinical raters (the reference-standard). We analysed data from 60 subjects (990 smartphone tests, 2628 blinded video MDS-UPDRS III subitem ratings). Subject-level predictive performance was quantified as the leave-one-subject-out cross-validation (LOSO-CV) accuracy. A pre-specified analysis classified 70.3% (SEM 5.9%) of subjects into a similar category to any of three blinded clinical raters and was better than random (36.7%; SEM 4.3%) classification. Post hoc optimisation of classifier and feature selection improved performance further (78.7%, SEM 5.1%), although individual subtests were variable (range 53.2–97.0%). Smartphone-based measures of motor severity have predictive value at the subject level. Future studies should similarly mitigate against subjective and feature selection biases and assess performance across a range of motor features as part of a broader strategy to avoid overly optimistic performance estimates.

## Introduction

Precise and sensitive measures of the motor severity of Parkinson’s disease (PD) remain elusive but critical if new therapies are to be fairly and quickly evaluated. The internationally validated, familiar and easily interpretable Movement Disorder Society-Unified Parkinson’s Disease Rating Scale Part III (MDS-UPDRS III)^1^ continues to be the favoured primary endpoint of major trials in PD^2^. But it is time-consuming for clinicians and its poor calibration and sensitivity^3^ may have played a part in the gross failure of novel therapies to translate into clinical practice^4,5^.

Mobile devices, such as wearables and smartphones, enable low-cost objective *repeated* monitoring of motor severity that are likely to improve the sensitivity of clinical trials in which they are used as end-points. Digital assessments show promise^6–8^ but have yet to be widely adopted partly due to the lack of transparency and harmonisation of the analysis methods used and the lack of subsequent high-quality subject-level evidence comparing them to current reference-standard measures^9^.

Feasibility studies have reported that digital assessments correlate with total MDS-UPDRS III^8^ and subcomponents of it^6,10^ but the equivalence of digital and clinical measures at the *individual* level remains unclear. Cross-validated subject-level prediction of disease category and total MDS-UPDRS III has been demonstrated in ten patients^7^, but when scaled to larger numbers, a model discriminating disease category—the simpler task—was only accurate at the cost of unstable features^11^.

This failure to generalise may be due to three fundamental reasons. First, digital models trained with single human scores incorporate *subjective bias* within them, rather than removing it. Second, approaches that consist of only 5–7 digital subtests may be too blunt to capture the *individual heterogeneity*^12^ evident within the 33-item clinical MDS-UPDRS III, and third, previous studies are at high risk of providing over-optimistic results due to *feature selection bias* if a large number of post hoc candidate digital features or machine-learning algorithms are tested within a limited size study.

We designed the CloudUPDRS Smartphone Software in Parkinson’s (CUSSP) study to address these concerns. We assessed the degree to which subject-level smartphone-based measures predicted subject-level MDS-UPDRS III subitems. A randomised crossover design and blinded assessment by three clinical raters mitigated concerns about subjective bias. We used a larger 16-item smartphone-based assessment to increase the capacity to capture individual heterogeneity, and methods were pre-published^6^ or pre-registered to reduce post hoc feature selection bias.

## Results

### Cohort details

**Fig. 1:**
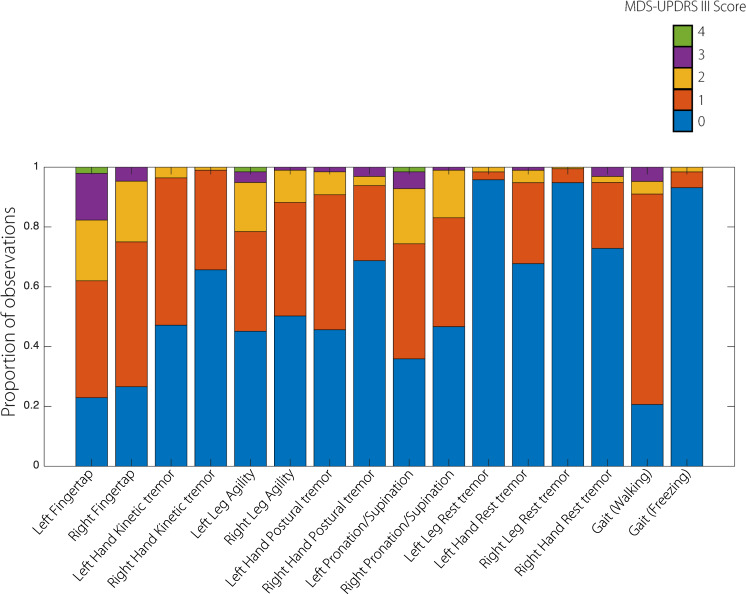
Clinical MDS-UPDRS III score subitem distributions. Distributions show a skew towards lower values consistent with other studies of early and moderate disease. Some clinical features (notably Left and Right Leg Tremor) show poor variation within this sample of 60 individuals with Parkinson’s.

Overall, 62 participants were recruited with a minority (5) from the second site (Homerton University Hospital), which started recruitment later. Network faults during data capture and storage errors of video-recordings, or incorrect task performance (detected on review of video-recordings) resulted in loss of data for two subjects, 18/1008 remaining smartphone tests and 6/882 remaining clinical MDS-UPDRS III items. Only three participants opted for an OFF/ON recording, the treatment status of the remaining participants being considered intermediate. Subsequently, the final analysis included 60 patients, 63 sessions, 990 smartphone subtests, 876 MDS-UPDRS III item videos and 2628 human score ratings. Twenty participants were female, eight participants were left-handed. Demographic details are shown in Table 1 and confirm that patients in this cohort were wide-ranging in age, had prominent motor and non-motor symptoms, required moderate amounts of medication and tended to remain functionally independent without cognitive impairment (as selected by the inclusion criteria). The total MDS-UPDRS III scores were skewed towards the mild/moderate end, as expected given the inclusion criteria. The subitems showing the greatest variation were left finger tapping and left pronation/supination, whilst left and right leg tremor showed very little variation (see Fig. 1). There was significant inter-rater agreement between clinical raters in all subitems at the population level, with rates of agreement mostly ranging from moderate to substantial (apart from left pronation/supination where agreement was only fair, see Table 2). Subject-level inter-rater agreement analysis showed that all three blinded clinical raters rarely completely disagreed (<5%, see Table 2).

**Table 1:** Demographic details of the cohort.

		Range	
	Median	5th percentile	95th percentile
Age (years)	68	51.6	80
Disease duration (years)	5	1	17
Levodopa equivalent dose (mg/day)	452	100	1345.6
Montreal Cognitive Assessment (range = 0–30)	27	21.6	30
Beck Depression Inventory (range = 0–63)	9	1.6	25.8
PDQ-39 (range = 0–100)	32	6.6	77.9
Hoehn & Yahr (range 1–5)	2	2	2
MDS-UPDRS III (0–132)^a^	31.5	10.2	50.8

Note that minimum Hoehn & Yahr stage was 1, the maximum was 4, but the frequency of these were low in this group.

^a^The total MDS-UPDRS III score provided is from the clinical assessor who performed the examination. Replacing individual subject subitem scores with the median of their counterpart blinded video ratings, changes the MDS-UPDRS III total population median to 26.5 (range 12–46).

**Table 2:** Population-level and subject-level inter-rater agreement for clinical MDS-UPDRS III subscores.

Subtest	Number of categories	Kappa (SEM)	95% confidence interval	Agreement	*p* value	Subject-level agreement (a/b/c)
Left hand rest tremor	4	0.68 (0.06)	0.65−0.71	Substantial	<0.00001	96.7/1.6/1.6
Right hand rest tremor	4	0.62 (0.06)	0.58−0.65	Substantial	<0.00001	72.6/27.4/0
Left leg rest tremor	3	0.58 (0.07)	0.54−0.61	Moderate	<0.00001	93.5/6.45/0
Right leg rest tremor	3	0.72 (0.06)	0.69−0.75	Substantial	<0.00001	82.5/17.5/0
Left hand postural tremor	4	0.76 (0.06)	0.73−0.79	Substantial	<0.00001	79.3/20.6/0
Right hand postural tremor	4	0.75 (0.06)	0.72−0.78	Substantial	<0.00001	82.5/17.5/0
Left hand kinetic tremor	3	0.62 (0.07)	0.59−0.66	Substantial	<0.00001	69.8/30.2/0
Right hand kinetic tremor	3	0.45 (0.07)	0.41−0.49	Moderate	<0.00001	61.9/38.1/0
Left fingertap	5	0.54 (0.04)	0.52−0.56	Moderate	<0.00001	50.0/48.4/1.6
Right fingertap	4	0.64 (0.05)	0.61−0.66	Substantial	<0.00001	64.5/35.5/0
Left pronation/supination	5	0.42 (0.05)	0.40−0.44	Moderate	<0.00001	42.9/52.4/4.8
Right pronation/supination	4	0.37 (0.05)	0.34−0.39	Fair	<0.00001	42.9/52.4/4.8
Left leg agility	5	0.55 (0.05)	0.53−0.58	Moderate	<0.00001	57.1/41.3/1.6
Right leg agility	4	0.57 (0.06)	0.54−0.60	Moderate	<0.00001	61.9/38.1/0

The population-level inter-rater agreement for each MDS-UPDRS III subscore was calculated using Fleiss’ Kappa. This ranges from −1 to 1, where 0 indicates chance agreement, 1 indicates complete agreement, and −1 indicates complete disagreement. Kappa values are shown for each subtest along with standard error of the mean (SEM) and 95% confidence intervals. The number of categories available in the sample (of all raters) is also shown. All estimates of agreement were highly significant. The subject-level agreement was calculated as the percentage of subjects where the blinded rating clinicians (a) agreed completely (3 raters agreed), (b) agreed moderately (2 raters agreed) or (c) disagreed (all 3 ratings were different). This is shown as a/b/c in the table above. Complete disagreement was rare (<5% in all subitems).

### Overall predictive accuracy of smartphone assessments

**Fig. 2:**
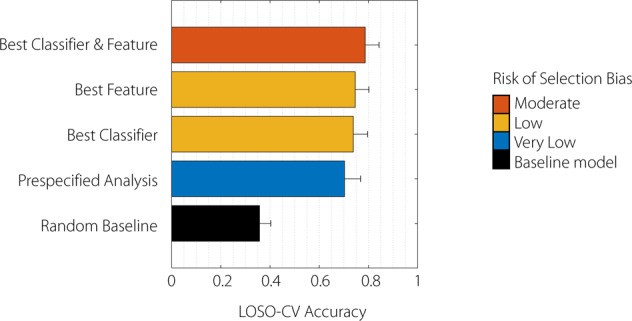
Primary outcome (any-rater criterion). The mean leave-one-subject-out cross-validation (LOSO-CV) classification accuracy of the smartphone-based prediction of the blinded MDS-UPDRS III. The accuracy of a number of approaches is compared to a random baseline (similar to rolling a dice where subjects were randomly assigned to a clinical category). The fully pre-specified analysis (blue) relied on pre-published features and a standard multinomial regression model. The Best Classifier approach selected the best classifier from a range based on best performance but used only the pre-specified features. The Best Feature approach selected the best feature from a range but used only the pre-specified classifier. The Best Classifier and Feature approach selected the best combination of both. Approaches are graded according to the risk of selection bias: the pre-specified analysis has a very low risk, the Best Classifier or Best Feature analyses have low risk whilst the combination approach has a moderate risk of over-optimistic accuracy. Error bars represent SEM.

The primary outcome was the overall leave-one-subject-out cross-validation (LOSO-CV) classification accuracy of the smartphone-based prediction of the MDS-UPDRS III subscores. A fully pre-specified analysis classified 70.3% (SEM 5.9%) of subjects into a similar category to a clinical rater (see Fig. 2 and Supplementary Table 4). This was above a random (36.7%; SEM 4.3%) baseline and below the performance achieved with optimised classifier and feature selection (78.7%, SEM 5.1%). Classifiers generally predicted more than one category with notable exceptions where there was an extreme degree of class imbalance within the clinical scores (left or right leg tremor, left hand kinetic tremor; see Supplementary Table 4). Smartphone scores were poorer at predicting the *median* MDS-UPDRS III subscores overall (57.0%, SEM 8.0%) but this was improved with optimised classifier and feature selection (65.2%, SEM 7.5%; see Supplementary Table 5).

### Item-specific predictive accuracy of smartphone assessments

**Fig. 3:**
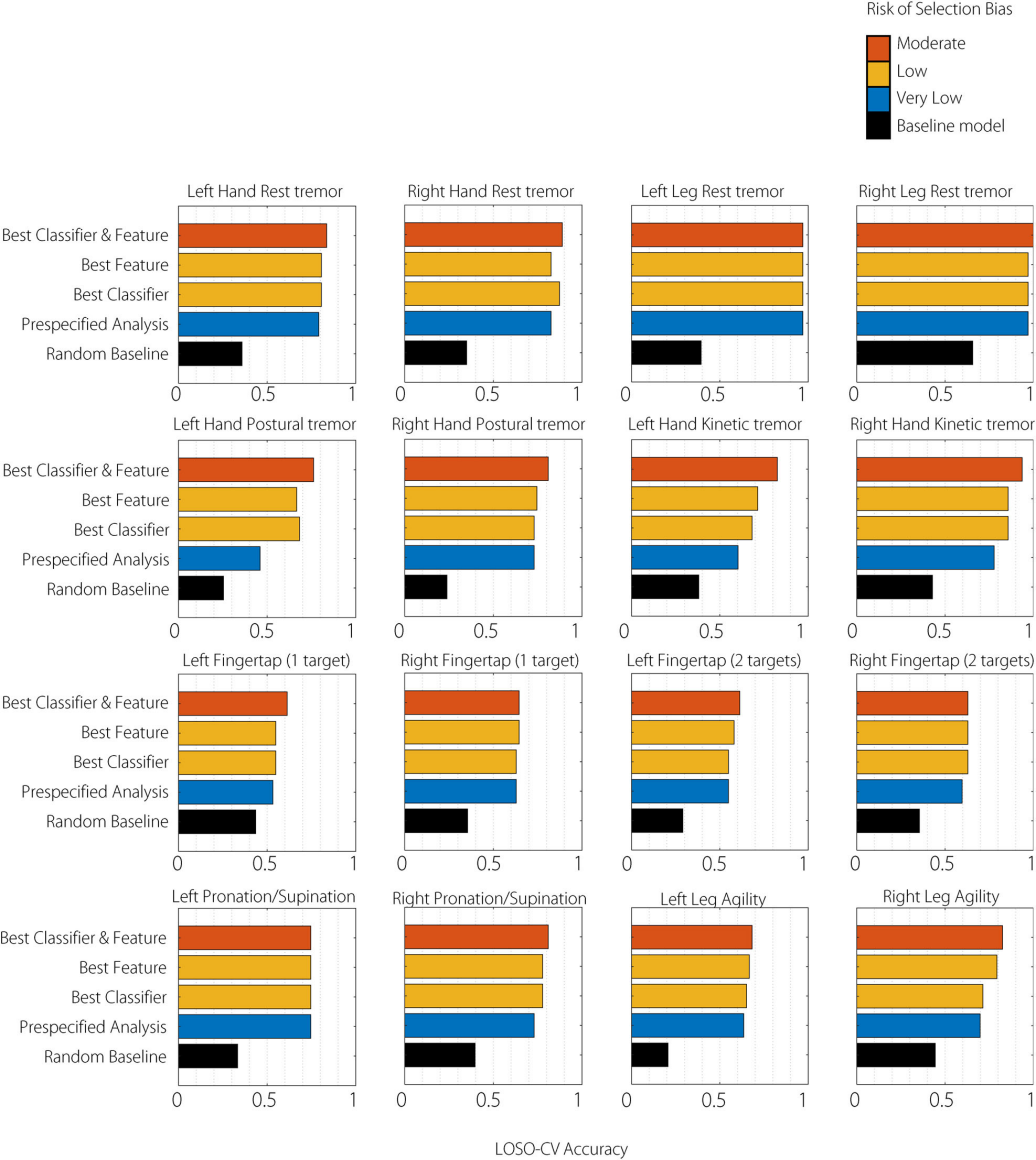
Individual Test Prediction Accuracy (any-rater criterion). The leave-one-subject-out cross-validation (LOSO-CV) classification accuracy of individual subtest smartphone-based prediction of the blinded MDS-UPDRS III subitems. Baselines and models are described in the legend to Fig. 2. Subtests relate to the corresponding MDS-UPDRS III subitems. Note that the Finger tapping MDS-UPDRS III subitem is repeated because it is predicted by two smartphone tasks (one or two target tapping).

Item-specific LOSO-CV accuracy for each of the 16 smartphone tests is shown in Fig. 3 and Supplementary Table 4. Notably although classifier performance for tremor was universally high, in the case of leg tremor (left leg tremor 97.0%, right leg tremor 97.0%), this was achieved by simply predicting the commonest category consistently (very few participants had leg tremor). On the other hand, bradykinesia scores had good variation across subjects and the highest pre-specified analysis accuracies were achieved for bilateral pronation/supination movements (left 74.6%, right 73.0%) and bilateral leg agility (left 63.5%, right 69.8%) rather than variants of finger tapping (1 target variant: left 53.2%, right 62.9%). The median-rater item-specific agreement analysis followed a similar pattern but with generally lower accuracy values; see Supplementary Table 5. The best-performing classifiers for each were as follows: Radial Basis Function Support Vector Machine for left hand rest tremor, right pronation/supination; AdaBoost for right hand rest tremor, left fingertap (1 and 2 targets), right/left leg agility; Nearest Neighbours for left leg rest tremor; Decision Tree for right leg rest tremor, left hand postural tremor, and left/right hand kinetic tremor; Random Forest for right hand postural tremor; Naïve Bayes for right fingertap (1 target); Linear Support Vector Machine for right fingertap (2 targets); Multinomial Logistic Regression for left pronation/supination (see Supplementary Tables 2 and 3 for full details).

## Discussion

New measures of motor severity in PD must be robustly evaluated at the subject level prior to widespread use. Although digital assessments such as finger tapping speed are objective and more likely to be reproducible, they can only be meaningfully interpreted as severity scales either when matched to prima facie valid subject-level outcomes such as degree of functional dependence or, more commonly, when matched to well-established and familiar but subjective scales such as the MDS-UPDRS III. Subsequently, most digital tools trained on single human classifications or ratings incorporate the subjective bias of that particular rater within them^6,8,10^. Here, we mitigate against this bias by training on the median of three blinded human ratings with mostly moderate-to-substantial inter-rater agreement (see Table 2), reducing the potential idiosyncratic influence of any individual rater (i.e. over-fitting to a particular rater). This should improve the subsequent generalisability of the smartphone-based measures that have been developed here. We report that even when generalised to out-of-sample subjects, classification of CloudUPDRS smartphone measures was reasonably equivalent to at least one of three MDS-UPDRS III human-raters overall (LOSO-CV accuracy 70.3%; SEM 5.9%), and that individual subtests had variable degrees of correspondence (Fig. 3 and Supplementary Table 4). A more stringent analysis requiring smartphone-derived measures to predict the median of three clinical raters exactly (a requirement to perform *better* than any individual human rater) was as expected less accurate overall (57.0%, SEM 8.0%) but still better than a random baseline (28.5%, SEM 4.7%).

Any clinical study is limited by how well the study cohort represents the clinical population in question. We analysed data from 60 subjects which cannot represent the entire population but compares favourably with other studies comparing supervised smartphone measures to MDS-UPDRS III scores^7,8,10^. Our selection criteria successfully captured those with mild to moderate disease who may have mild/moderate depression or mild cognitive impairment (MoCA > 20), but not of a severity that would impair understanding of the simple motor tasks required. The sample is, therefore, broader and more transparent than previous reports and more representative of a typical secondary care population, recruiting from two hospital sites and not excluding patients based on poor compliance with home monitoring, or phone availability for example. In spite of this, some clinical features (e.g. leg tremor) did not have enough sample variation to train a useful predictive model, and generally models were more sensitive to changes in mild to moderate (rather than severe) disease. This highlights an important limitation to most current digital studies that focus on mild/moderately affected individuals—if classifiers are not trained on data from more severely affected patients (or patients assess in the ‘OFF’ state), they will not generalise well to these populations. In our study, only three subjects opted to be assessed after overnight withdrawal from dopaminergic medication. Although the effect of treatment is not the question we are addressing here, this may have limited the number of high MDS-UPDRS III scores in our cohort. Similarly to other digital tools, therefore, our smartphone software may be of most benefit to populations with mild/moderate disease undergoing interventions to modify disease progression or treat motor fluctuations. Future work should aim to address this by collecting more data or changing inclusion criteria to incorporate more severely affected patients. Because all assessments were performed under clinical supervision and reviewed by video, we can also have confidence that the smartphone tasks were performed adequately without undue influence from idiosyncratic factors (such as inadequate understanding of the tasks, concurrent disabling dyskinesias or distraction by a conversation). Although mild cognitive impairment and depression may correlate with disease severity and therefore slower movements, this is likely to equally affect the clinical MDS-UPDRS III and smartphone subtests and therefore is unlikely to be a significant confound to the primary outcome. Finally, the reproducibility of these findings also depends on the number of blinded clinical raters and their inter-rater agreement. Our three blinded raters were of similar clinical experience and so population-level and subject-level inter-rater agreement was reasonable. Future work may look at the effect of a larger and more varied pool of trained blinded raters.

PD is heterogeneous—the motor signs of tremor and bradykinesia typically vary within a patient across body parts and over time^12^. Most current digital assessments that focus on only 5–7 pre-selected subtests^13–17^ are likely to have reduced sensitivity as compared to the 33-item MDS-UPDRS III. We rely on 16 independent smartphone subtests to evaluate individual heterogeneity across body parts and report results for individual tests in addition to a combined score (see Figs. 2 and 3 and Supplementary Table 4). This allows for a closer match to the original MDS-UPDRS III, but some tests (notably of rigidity and axial features) remain currently outwith the scope of smartphone tests. Motor features such as tremor amplitude can change over minutes and so we explicitly randomised the order of smartphone and clinical assessments (performed within minutes of each other) across patients to avoid systematic biases in measurement present in other studies. We found that clinical tremor scores were predicted well by subject-level smartphone measures (LOSO-CV accuracy 46−97%) in keeping with its importance in previous studies^18,19^, but that in the case of leg tremor, this was largely due to low variation in the sample of MDS-UPDRS III tremor scores. Without collecting additional functional outcome data, however, it remains unclear whether this apparent insensitivity of the MDS-UPDRS III tremor classification has a clinically meaningful significance. Bradykinesia tests also showed variable performance with pronation/supination measures standing out as having good sampling variance across the population and good subject-level correspondence between clinical and digital measures (mean LOSO-CV accuracy 73–74.6%). This is an important consideration for current smartphone assessments that typically focus on finger-tapping only^13–16^. Future analyses should determine whether a combination of subtests can be used to predict non-tested items (such as limb rigidity) or approximate total MDS-UPDRS III score but this is beyond the scope of the pre-specified analysis plan that we report here. Until this is established, we suggest ongoing assessment of a broader range of motor tests to improve the sensitivity of subject-level smartphone-based measures to diverse motor presentations of disease.

Conversion of a raw digital measurement into a useful digital biomarker typically requires specific data selection and transformation (feature selection) and choice of an optimal machine-learning algorithm. The greater the pool of post hoc features and algorithms from which the selection takes place, the greater the risk of *feature selection bias* where predictive accuracy is over-optimistic and relies on chance relations in the data (also known as ‘over-fitting’). This problem is magnified when the ratio of features to observations is high and may explain why previous models failed to generalise^11^. We mitigated against this by pre-registering our design and features^6^ and using a standard linear classification algorithm. Our approach, based on more restrictive pre-specified analysis and the consideration of individual features only, is conservative compared to the majority of prior studies in the literature. We additionally performed an exploratory graded feature and classifier selection process which maximally led to an improved overall LOSO-CV accuracy of 78.7% (SEM 5.1). The combined results provide conservative and relatively unbiased benchmark accuracies suitable for clinical translation (full pre-specification) together with increasingly optimised results which are at risk of bias, but facilitate comparison with other studies and suggest optimal features and classifiers to be tested by future research.

This study demonstrates how digital assessments for PD can be robustly validated within prospective clinical trials—a necessary step prior to widespread adoption. We mitigated against prior concerns of subjective bias and feature selection bias whilst remaining sensitive to the diverse motor presentations of the condition and utilising open-source analysis software. Our evaluation provides a conservative but relatively unbiased benchmark performance of how smartphone-based measures correspond to standard-reference measures at the subject level. Future smartphone-based studies should consider similar precautions and incorporate more severely affected patients to improve the reliability and generalisability of their conclusions.

## Methods

### Study design

We designed and conducted a prospective, pre-registered (NCT02937324), dual-site crossover-randomised study comparing structured single time-point smartphone-based and blinded clinical rater assessments of motor severity in PD. The primary outcome was the degree to which subject-level smartphone-based measures predicted subject-level MDS-UPDRS III as calculated by three blinded clinical raters. This was quantified as the leave-one-subject-out cross-validation (LOSO-CV) predictive accuracy. This work has received ethical approval from the UK HRA and the local research ethics committee and written informed consent was obtained from all participants.

### Participants

Participants were recruited from both the National Hospital for Neurology and Neurosurgery, London, UK and Homerton University Hospital, London, UK between 8 August 2017 and 1 March 2019. The inclusion criteria were (1) diagnosis of probable idiopathic PD according to Brain Bank criteria^20^, (2) age over 18 years old, (3) a score >20/30 on the Montreal Cognitive Assessment (MoCA)^21^, (4) no anti-parkinsonism medication changes within the last week, (5) capacity to consent, (6) ability to understand English well enough to operate the phone software, follow its instructions and be able to answer the study questions. Exclusion criteria were: (1) concurrent acute medical illness, (2) other co-morbidity that in the opinion of the Investigator may preclude their participation in the study, (3) inability to consent. A target of 60 participants was pre-specified based on feasibility rather than a power calculation.

### Clinical data collection

Patient data were collected over one or two visits, as individually convenient. Screening assessments performed after enrolment included the full MDS-UPDRS^1^, MoCA^21^, Beck Depression Inventory^22^, PDQ-39^23^ and Hoehn and Yahr^24^. Participants opted to perform the motor assessments immediately, or at a second morning visit after overnight withdrawal of their anti-parkinsonian medications. Participants attending for a second visit performed the test initially in a practically defined ‘OFF’ state, and then took their usual medication and repeated the testing in the ‘ON’ state. The latter test was carried out after a maximum of 1.5 h from the initial testing and only after both the examiner and participant agreed that the participant had entered the ‘ON’ state. Participants attending for only one visit were labelled as an ‘Intermediate’ state.

Each motor assessment included a standard video-recorded assessment of the 33-item MDS-UPDRS III^1^ by a member of the movement disorder clinical team and a 16-item smartphone-based assessment supervised by the clinician. Smartphone assessments were performed on either the participant’s own phone or on a smartphone supplied by the study team depending on availability. The smartphone and clinical assessments were performed one immediately after the other in a crossover design—their order randomised by software within the CloudUPDRS application at the point of enrolment for each participant.

### Blinded clinical video evaluation

In addition to the live clinical MDS-UPDRS III ascertained by the examining clinician, we also obtained three blinded rater scores to mitigate against subjective rater bias. Three neurologists with at least 12 months of specialist movement disorders training post certification who were not involved with the initial data collection, separately rated the *videoed* examination of each MDS-UPDRS III subcomponent included in the study (3.4 Finger tapping, 3.6 Pronation/supination movements of hands, 3.8 Leg agility, 3.15 Postural tremor of hands, 3.16 Kinetic tremor of hands, 3.17 Rest tremor amplitude; see Supplementary Table 1 for further details) blinded to the medication status, crossover randomisation order and clinical details of the participant and to each others’ scores.

### The CloudUPDRS system

The CloudUPDRS system is a CE marked software device consisting of the CloudUPDRS application for Android smartphones, a cloud-based scalable data-collection service and a data-mining toolkit that we developed (Manufacturer: Birkbeck College, University of London, MHRA Manufacturer’s Ref. number CA015327, Class 1a Medical Device, date: 29/2/16)^6,25,26^. The graphical-user-interface has been iteratively improved over a number of design cycles with the input of patient-led focus groups, clinicians and user-interface design experts to improve user experience in the target population, many of whom have impaired visual perception and dexterity. As far as possible it is also designed to harmonise data collection across multiple device types (e.g. by preserving distance and size of tapping targets)^26^. The software guides each participant through 17 subtests that correspond to subcomponents of the MDS-UPDRS III. Each subtest has specific written, visual and audio instructions (available at http://www.updrs.net/help/) and lasts between 60 and 90 s during which time the relevant phone sensor is recorded at the maximum sampling rate the phone allows (minimum of 50 Hz). Raw sensor data are time-stamped, assigned a unique identification number and stored locally. On completion of the test subsets, data are automatically uploaded to a remote secure server via an encrypted link. Full computational details have been published previously^25,26^.

### Smartphone data collection

Smartphone subtests were designed to resemble the MDS-UPDRS III as far as possible, whilst also considering safety and practicality in the context of concurrent smartphone use. Three types of subtests were performed. Finger tapping required participants to tap one or two consistently spaced targets on the smartphone whilst screen sensors recorded the onset, duration, upwards or downwards movement and the coordinates of each touch. Tremor and proximal limb bradykinesia tests required the participant to hold or repeatedly move the phone in a stereotyped pattern whilst acceleration in 3- or 6-axes was recorded. Walking tests required the participant to place the phone in their pocket and walk 5 m, turn around and walk back (however note that gait analysis was not part of the pre-specification and so has been excluded from the current report). Supplementary Table 1 shows the correspondence between each MDS-UPDRS III item and each CloudUPDRS subtest, and gives details of sensors used, recording times and the features extracted. Note that the clinical MDS-UPDRS III subitem Finger tapping is assessed with two separate smartphone test items per hand (One Target Finger Tapping and Two Target Finger Tapping). Participants were not given any specific training and were asked to follow the onscreen instructions when performing smartphone tasks. In some cases, examiners emphasised the aspects of the instruction to ensure the task was performed correctly (e.g. ‘with your *left* hand first’, ‘tap as *fast* as you can for 1 min’). All smartphone examinations were also video-recorded and reviewed to confirm correct task performance. Trials where there were substantial errors, such as unintended use of wrong hand, were removed.

### Smartphone data preprocessing and feature extraction

Raw data were stored in a custom flat text files and processed offline using PDkit version 1.2.1 (https://github.com/pdkit/pdkit), an open-source data science toolkit for PD running in a Python 3 environment that has been previously developed by some of the authors of this study. Data were ingested and converted to a standardised time-series data type. There are many ways in which subject-level acceleration time-series data and touch event data can be summarised by single numbers (features). For example, acceleration data can be characterised by the power at its peak frequency or the amplitude over a frequency range. Touch event data can be summarised as a function of the hold-time of a tap, the time between taps or the spatial proximity of the touch to the visual target. PDKit provides robust, transparent and automated extraction of hundreds of distinct features currently in use in the literature and is designed to support harmonisation and comparison of metrics within one fully transparent and non-proprietary platform. We calculated nine features for finger tapping subtests, and 35 features for each other subtest. A persistent archive of feature names and the code used to calculate them is available at: 10.5281/zenodo.3632529.

### Pre-specified features

We separately report analyses of pre-specified features (from our previously reported study^6^) and the best-performing feature for each subtest (see below). Pre-specified features were calculated exactly as previously. That is, for tremor subtests, the magnitude of the scalar sum acceleration in three axes was filtered with a high-pass second-order Butterworth filter at 2 Hz prior to fast Fourier transform. Tremor amplitude was calculated as the sum of the resultant power spectrum between 2 and 10 Hz. For bradykinesia assessments excluding tapping tests, the signal had DC removed prior to applying a low-pass second-order Butterworth filter at 4 Hz and subsequent fast Fourier transform. The amplitude of the remaining signal was calculated as the sum of the amplitude between 0 and 4 Hz. Note that in this context, amplitude relates to the ‘magnitude’ of tremor in the frequency domain rather than the measured distance travelled during movement. For single- or dual-tapping tests we calculated the tapping frequency as the total number of taps divided by the time period of the task (60 s).

### Graded approach to mitigating feature and classifier selection bias

With many more features available than observations, post hoc selection of one or a few of these features is a commonly used strategy to improve model stability. This can, however, subsequently induce feature selection bias. Post hoc selection of a classifier can induce a similar bias and so we adopted a graded approach to address both issues. At the conservative end, we used single pre-specified features from our previously reported study^6^ and standard statistical classifiers (multinomial logistic regression) maximally free from bias but likely to under-fit the data (under-optimistic accuracy). At the exploratory end, we selected both the feature and classifier with maximum out-of-sample predictive accuracy which is moderately likely to over-fit the data (over-optimistic accuracy). Intermediate accuracy values where either the classifier or the feature were selected based on performance are also presented. A full description of the feature and classifier selection process and details of the pre-specified features are presented in Supplementary Note 2. Additionally, all available features and the accompanying software implementation can be viewed in the PDkit online documentation (https://pdkit.readthedocs.io/).

### Statistical analysis

The primary outcome was the overall LOSO-CV classification accuracy of the smartphone-based prediction of selected subitems of the MDS-UPDRS III (3.4 Finger tapping, 3.6 Pronation/Supination movements of hands, 3.8 Leg agility, 3.15 Postural tremor of hands, 3.16 Kinetic tremor of hands, 3.17 Rest tremor amplitude). For each subject, each item from the MDS-UPDRS III was blindly rated three times and so the median of these clinical ratings was taken forward as the dependent variable for model training. For each of the 16 smartphone subtests, a single (normalised) smartphone feature was entered into a classifier using the corresponding median MDS-UPDRS III subscore as the target variable. The target score maximally consisted of five categories reflecting the ordinal 5-point scale of each MDS-UPDRS III item, and often consisted of fewer categories being restricted to measures in the study population sample. To reduce the risk of over-fitting and to understand how classifiers would perform on new unseen subjects, we performed LOSO-CV. The out-of-sample prediction for each subject was therefore made from a model trained only on the remaining data. Correct classification was defined as an individual prediction consistent with any of the three individual clinical raters (any-rater criterion). We additionally performed a more stringent analysis in which correct classification was defined as an individual prediction consistent with the median of three individual clinical raters (median-rater criterion). Higher accuracy can reflect characteristics of the target distribution (the distribution of MDS-UPDRS III item scores) such as class imbalance, rather than the utility of the smartphone data and so we performed two additional checks. First, we determined whether the pre-specified models were predicting a range of categories rather than consistently predicting one category. Secondly, we provide a reference ‘random baseline’ accuracy from a classifier that randomly assigns subjects into each available clinical category with uniform probability across categories. As well as using pre-specified features and the standard multinomial logistic regression model, we tested a number of other feature and classifier combinations using the same LOSO-CV procedure (see Supplementary Note 2).

To ensure the three blinded clinical raters were consistent with each other, we additionally calculated inter-rater reliability for each MDS-UPDRS III subscore using Fleiss’ Kappa, which is a generalisation of Cohen’s Kappa to more than two raters^27^. Kappa ranges from −1 to 1, where 0 indicates chance agreement, 1 indicates complete agreement, and −1 indicates complete disagreement at the population level. We also provide a measure of subject-level inter-rater agreement. This was calculated for each MDS-UPDRS III subscore as the percentage of subjects where the blinded rating clinicians agreed completely (3 raters agreed), moderately (2 raters agreed) or disagreed (all 3 ratings were different). Inter-rater agreement was calculated in Matlab R2020a (The MathWorks Inc, Natick, MA, USA) using the Fleiss toolbox (https://www.github.com/dnafinder/Fleiss).

### Reporting summary

Further information on research design is available in the Nature Research Reporting Summary linked to this article.

## Supplementary information

Supplementary Material

reporting summary

## Data Availability

Anonymised summary data that support the findings of this study are available from the corresponding authors upon reasonable request.

## Code availability

PDkit version 1.2.1 was used to process the smartphone data features and is available at https://github.com/pdkit/pdkit. Classification was performed using the scikit-learn toolbox version 0.22 available at https://scikit-learn.org.

## References

[1] Goetz CG (2008). Movement Disorder Society-sponsored revision of the Unified Parkinson’s Disease Rating Scale (MDS-UPDRS): scale presentation and clinimetric testing results. Mov. Disord..

[2] Verschuur CVM (2019). Randomized delayed-start trial of Levodopa in Parkinson’s disease. N. Engl. J. Med..

[3] Regnault A (2019). Does the MDS-UPDRS provide the precision to assess progression in early Parkinson’s disease? Learnings from the Parkinson’s progression marker initiative cohort. J. Neurol..

[4] Athauda D, Foltynie T (2015). The ongoing pursuit of neuroprotective therapies in Parkinson disease. Nat. Rev. Neurol..

[5] Dorsey ER, Papapetropoulos S, Xiong M, Kieburtz K (2017). The first frontier: digital biomarkers for neurodegenerative disorders. Digit. Biomark.

[6] Kassavetis P, Saifee TA, Roussos G, Drougkas L, Kojovic M (2015). Developing a tool for remote digital assessment of Parkinsonas disease. Mov. Disord. Clin. Pract..

[7] Arora S (2015). Detecting and monitoring the symptoms of Parkinson’ s disease using smartphones: a pilot study. Park. Relat. Disord..

[8] Zhan A (2018). Using smartphones and machine learning to quantify Parkinson disease severity the mobile Parkinson disease score. JAMA Neurol..

[9] Espay AJ (2019). A roadmap for implementation of patient-centered digital outcome measures in Parkinson’s disease obtained using mobile health technologies. Mov. Disord..

[10] Lipsmeier F (2018). Evaluation of smartphone-based testing to generate exploratory outcome measures in a phase 1 Parkinson’s disease clinical trial. Mov. Disord..

[11] Arora S (2018). Smartphone motor testing to distinguish idiopathic REM sleep behavior disorder, controls, and PD. Neurology.

[12] Greenland JC, Williams-Gray CH, Barker RA (2019). The clinical heterogeneity of Parkinson’s disease and its therapeutic implications. Eur. J. Neurosci..

[13] Lee, C. Y. et al. A validation study of a smartphone-based finger tapping application for quantitative assessment of bradykinesia in Parkinson’s disease. *PLoS ONE* **11**, e0158852, 10.1371/journal.pone.0158852 (2016). 10.1371/journal.pone.0158852 PMC4965104 27467066

[14] Heldman DA, Espay AJ, LeWitt PA, Giuffrida JP (2014). Clinician versus machine: reliability and responsiveness of motor endpoints in Parkinson’s disease. Park. Relat. Disord..

[15] Bot BM (2016). The mPower study, Parkinson disease mobile data collected using ResearchKit. Sci. Data.

[16] Hasan H (2019). The BRadykinesia Akinesia INcoordination (BRAIN) tap test: capturing the sequence effect. Mov. Disord. Clin. Pract..

[17] Printy, B. P. et al. Smartphone application for classification of motor impairment severity in Parkinson’s disease. *Annu. Int. Conf. IEEE Eng. Med. Biol. Soc.* **2014**, 2686–2689. 10.1109/EMBC.2014.6944176 (2014). 10.1109/EMBC.2014.6944176 25570544

[18] Kostikis, N., Hristu-Varsakelis, D., Arnaoutoglou, M. & Kotsavasiloglou, C. Smartphone-based evaluation of parkinsonian hand tremor: Quantitative measurements vs clinical assessment scores. *Annu. Int. Conf. IEEE Eng. Med. Biol. Soc.* **2014**, 906–909. 10.1109/EMBC.2014.6943738 (2014). 10.1109/EMBC.2014.6943738 25570106

[19] Bazgir O, Habibi SAH, Palma L, Pierleoni P, Nafees S (2018). A classification system for assessment and home monitoring of tremor in patients with Parkinson’s disease. J. Med. Signals Sens..

[20] Gibb WRG, Lees AJ (1988). The relevance of the Lewy body to the pathogenesis of idiopathic Parkinson’s disease. J. Neurol. Neurosurg. Psychiatry.

[21] Rossetti HC, Lacritz LH, Munro Cullum C, Weiner MF (2011). Normative data for the Montreal Cognitive Assessment (MoCA) in a population-based sample. Neurology.

[22] Beck AT, Steer RA, Carbin MG (1988). Psychometric properties of the Beck Depression Inventory: twenty-five years of evaluation. Clin. Psychol. Rev..

[23] Crispin, J, Fitzpatrick, R. and Peto, V. *Parkinson’s Disease Quality of Life Questionnaire (PDQ-39)* (Oxford University, 1993).

[24] Hoehn MM, Yahr MD (1967). Parkinsonism: onset, progression, and mortality. Neurology.

[25] Kueppers, S. et al. From wellness to medical diagnostic apps: the Parkinson’s disease case. In Giokas K., Bokor L., Hopfgartner F. (eds) *eHealth 360°. Lecture Notes of the Institute for Computer Sciences, Social Informatics and Telecommunications Engineering*, Vol. 181. Springer, Cham (2017).

[26] Stamate C (2018). The cloudUPDRS app: a medical device for the clinical assessment of Parkinson’s Disease. Pervasive Mob. Comput..

[27] Fleiss JL (1971). Measuring nominal scale agreement among many raters. Psychol. Bull..

