# Supplementary material for: The CloudUPDRS smartphone software in Parkinson’s study: cross-validation against blinded human raters
Source: NPJ Parkinsons Dis. 2020 Dec 8;6:36. doi: 10.1038/s41531-020-00135-w (PMC7722731; doi:10.1038/s41531-020-00135-w)
Supplement: Supplementary file 1 — Supplementary Material [file 41531_2020_135_MOESM1_ESM.pdf]

The CloudUPDRS Smartphone Software in Parkinson's study: Cross-validation against blinded human raters.

Ashwani Jha[1]*, Elisa Menozzi[1,2], Rebecca Oyekan[1,3], Anna Latorre[1], Eoin Mulroy[1], Sebastian R Schreglmann[1], Cosmin Stamate[4], Ioannis Daskalopoulos[4], Stefan Kueppers[4], Marco Luchini[5], John C. Rothwell[1], George Roussos[3], Kailash P. Bhatia[1]*

[1]Department of Clinical and Movement Neurosciences, UCL Queen Square Institute of Neurology, London, UK

[2]Department of Biomedical, Metabolic and Neural Sciences, University of Modena and Reggio Emilia, Modena, Italy

[3]Queen Square Movement Disorders Centre, Department of Clinical and Movement Neurosciences, UCL Queen Square Institute of Neurology, London, UK

[4]Birkbeck College, University of London, London, UK

[5]Benchmark Performance Ltd, Colchester, UK

Supplementary Material

Contents:

Supplementary Note 1: Relation between CloudUPDRS smartphone and MDS-UPDRS III items

Supplementary Note 2: Feature and Classifier selection

Supplementary Data 1: LOSO-CV Prediction analysis (any-rater criterion)

Supplementary Data 2: LOSO-CV Prediction analysis (median-rater criterion)

Supplementary Note 1: Relation between CloudUPDRS smartphone and MDS-UPDRS III items

The MDS-UPDRS III score is a 33 component scale (each component scored 0-4), distributed over 18 sections (labelled section 3.1, 3.2,… 3.18) designed to measure aspects of movement in a patient with PD. The CloudUPDRS smartphone application aims to measure a subset of 14 of these components (8 sections, 48% of total score) using 16 tests, which are outlined in Supplementary Table 1. Note that Finger tapping is assessed with two separate smartphone test items per hand. Gait is assessed by the smartphone application but because this was not included in our previously reported study[6], we were unable to pre-specify a feature and so this has been excluded from the current study.

MDS-UPDRS III Item	Components	Smartphone subtest	Phone Sensors used	Test duration (s)	Example Features
3.4 Finger tapping	2	One target tapping	screen pressure and touch events and co-ordinates	60	Frequency, distance
3.4 Finger tapping	2	Two target tapping	screen pressure and touch events and co-ordinates	60	Frequency, distance
3.6 Pro/sup movements of hands	2	Pronation/ supination movements of hands	acceleration in 3- or 6- axes	30	Amplitude, frequency
3.8 Leg agility	2	Leg agility	acceleration in 3- or 6- axes	30	Amplitude, frequency
3.10 Gait	1	Gait	acceleration in 3- or 6- axes	90	Number of steps, stride length, speed
3.11 Freezing of gait	1	Freezing detection algorithm	acceleration in 3- or 6- axes	90	Gait speed variation
3.15 Postural tremor of hands	2	Postural tremor	acceleration in 3- or 6- axes	30	Tremor power (at dominant frequency)
3.16 Kinetic tremor of hands	2	Kinetic tremor	acceleration in 3- or 6- axes	30	Tremor power (at dominant frequency)
3.17 Rest tremor amplitude	4	Rest tremor	acceleration in 3- or 6- axes	30	Tremor power (at dominant frequency)

Supplementary Table 1: Correspondence between the clinician ascertained MDS-UPDRS III and the smartphone-based measures of motor severity. Each UPDRS section has 1-4 components, each scored from 0-4. For example, section 3.4 has 2 components because it is done individually in the left and right hand. Sections of part III of the motor UPDRS not included in the smartphone assessment are: 3.1 Speech, 3.2 Facial Expression, 3.3, Rigidity, 3.5 Hand movements, 3.7 Toe tapping, 3.9 arising from chair,

3.12 Postural instability, 3.13 Posture, 3.14 Global spontaneity of movement, 3.17 (rest tremor of jaw not included), 3.18 constancy of rest tremor.

Supplementary Note 2: Feature and Classifier selection

Post hoc selection of features and/or classifiers can induce feature selection bias and so we adopted a graded approach to address this. At the most conservative end, we used pre-specified features from our previously reported study[6] and standard statistical classifiers (multinomial logistic regression). We performed two intermediate analyses: the best performing classifer with pre-specified features and the best performing feature with a standard classifier. At the most exploratory end we selected the best performing feature and classifier combination. Best performance was determined for each feature or classifier as maximum LOSO-CV accuracy for each subtest. We did not pursue further exploratory analyses such as looking at multi-variable feature predictions or feature interactions as these approaches can suffer from a higher degree of feature selection bias.

For the univariable feature search, we used all the features available on the PDkit website. Across all 16 subtests, this amounted to 456 available features (at the time of analysis). Full details of all available features, their original source references and accompanying software implementation can be viewed in the PDkit online documentation (https://pdkit.readthedocs.io/).

All features were normalised with a Box-Cox transformation, that transforms the data into a truncated normal distribution to facilitate statistical analysis.

All LOSO-CV classification was performed using the scikit-learn toolbox version 0.22 (https://scikit-learn.org). A brief description of the algorithms used is provided in Supplementary Table 2.

Classifier	Description
Uniform	The prediction is randomly made from a uniform distribution over the categories available in the sample. Used to calculate the random baseline.
Multinomial Logistic Regression	A logistic function is used to map the multiclass ordinal outcomes onto the feature. The algorithm is solved using an optimisation procedure.
Nearest Neighbours	k-nearest neighbours was used where k was specified as the maximum number of categories in the available class.
Linear SVM	Linear Support Vector Machine using the one-against-one approach for multi-class classification[27].
RBF SVM	Radial Basis Function Support Vector Machine using the one-against-one approach for multi-class classification[27].
Gaussian Process	Gaussian process classification based on Laplace approximation based on Algorithm 3.1, 3.2, and 5.1 of Gaussian Processes for Machine Learning[28]. The kernel specifying the covariance function is set to RBF(1.0).
Decision Tree	A standard decision tree classifier with maximum depth set to 5.
Random Forest	A random forest meta estimator which employs several decision tree classifiers on sub-samples of the dataset and uses averaging to improve the predictive accuracy and control over-fitting. The number of trees of the forest is set to 10 and the maximum depth is set to 5 for each tree.
Neural Net	A Multi-layer Perceptron classifier optimising the log-loss function using a RELU activation function and the Adam solver for weight optimisation. The L2 penalty (regularisation term) parameter is set to 1.0 and 1,000 epochs for the Adam stochastic solver.
AdaBoost	A meta-estimator that first computes a classifier on the whole dataset and then proceeds to create copies of the classifier so that the weights of incorrectly classified instances are adjusted to favour more difficult cases. The implementation follows the Multi-class AdaBoost[29] by Zhu, Zou, Rosset and Hastie.
Naive Bayes	An implementation of the Gaussian Naive Bayes algorithm for classification following Chan, Golub, and LeVeque (Stanford CS tech report STAN-CS-79-773)

Supplementary Table 2: Description of classifiers used.

**Subtest**	**Prespecified Classifier & Feature**	**Best Classifier & Prespecified Feature**	**Prespecified Classifier & Best Feature**	**Best Classifier & Feature**
Left Hand Rest Tremor	Logistic Regression; *Amplitude by FFT*	Decision Tree; *Amplitude by FFT*	Logistic Regression; *Magnitude Spkt Welch De*	RBF SVM; *Magnitude Spkt Welch De*
Right Hand Rest Tremor	Logistic Regression; *Amplitude by FFT*	Neural Net; *Amplitude by FFT*	Logistic Regression; *Amplitude by FFT*	AdaBoost; *Magnitude Autocorrelation_lag_8*
Left Leg Rest Tremor	Logistic Regression; *Amplitude by FFT*	Nearest Neighbours; *Amplitude by FFT*	Logistic Regression; *Amplitude by FFT*	Nearest Neighbours; *Amplitude by FFT*
Right Leg Rest Tremor	Logistic Regression; *Amplitude by FFT*	Linear SVM; *Amplitude by FFT*	Logistic Regression; *Amplitude by FFT*	Decision Tree; *Frequency by Welch*
Left Hand Postural Tremor	Logistic Regression; *Amplitude by FFT*	RBF SVM; *Amplitude by FFT*	Logistic Regression; *Amplitude by Welch*	Decision Tree; *Amplitude by Welch*
Right Hand Postural Tremor	Logistic Regression; *Amplitude by FFT*	Linear SVM; *Amplitude by FFT*	Logistic Regression; *Magnitude Change Quant*	Random Forest; *Magnitude Agg Linear Tr*
Left Hand Kinetic Tremor	Logistic Regression; *Amplitude by FFT*	Nearest Neighbours; *Amplitude by FFT*	Logistic Regression; *Magnitude Number Peaks*	Decision Tree; *Frequency by Welch*
Right Hand Kinetic Tremor	Logistic Regression; *Amplitude by FFT*	Linear SVM; *Amplitude by FFT*	Logistic Regression; *Frequency by Welch*	Decision Tree; *Frequency by FFT*
Left Fingertap (1 target)	Logistic Regression; *Frequency*	Linear SVM; *Frequency*	Logistic Regression *Incoordination Score*	AdaBoost; *Mean Alnt Target Distan*
Right Fingertap (1 target)	Logistic Regression; *Frequency*	Linear SVM; *Frequency*	Logistic Regression; *Mean Alnt Target Distan*	Naïve Bayes; Mean Moving Time
Left Fingertap (2 targets)	Logistic Regression; *Frequency*	Linear SVM; *Frequency*	Logistic Regression; *Mean Moving Time*	AdaBoost; *Mean Moving Time*
Right Fingertap (2 targets)	Logistic Regression; *Frequency*	Linear SVM; *Frequency*	Logistic Regression; *Mean Moving Time*	Linear SVM; *Frequency*
Left Pronation/ Supination	Logistic Regression; *Amplitude by FFT*	Logistic Regression; *Amplitude by FFT*	Logistic Regression; *Amplitude by FFT*	Logistic Regression; *Amplitude by FFT*
Right Pronantion/ Supination	Logistic Regression; *Amplitude by FFT*	RBF SVM; *Amplitude by FFT*	Logistic Regression; *Amplitude by Welch*	RBF SVM; *Magnitude Mean*
Left Leg Agility	Logistic Regression; *Amplitude by FFT*	Neural Net; *Amplitude by FFT*	Logistic Regression; *Magnitude Agg Linear Tr*	AdaBoost; *Magnitude Agg Linear Tr*
Right leg Agility	Logistic Regression; *Amplitude by FFT*	Neural Net; *Amplitude by FFT*	Logistic Regression; *Magnitude Partial Auto*	AdaBoost; *Magnitude Partial Auto*

Supplementary Table 3: Features and classifiers used for each analysis. Features are italicised for convenience to separate them from classifiers which are not. Multinomial Logistic Regression has been abbreviated to Logistic Regression. For specifics of each feature, see https://pdkit.readthedocs.io/.

Supplementary Data 1: LOSO-CV Prediction analysis (any-rater criterion)

For the main analysis presented, we asked if the model predictions were similar to *any* other clinical rater. The any-rater criterion definition of a correct classification used is that for an individual prediction, the model was able to agree with any of the three individual clinical raters. Using this criterion, the following LOSO-CV accuracies were obtained (see also Figure 1 and Figure 2).

Subtest	Random Baseline	Prespecified Classifier & Feature	Prespecified Classifier & Feature categories predicted	Best Classifier & Prespecified Feature	Prespecified Classifier & Best Feature	Best Classifier & Feature
Left Hand Rest Tremor	35.8	79.1	3/4	80.6	80.6	83.6
Right Hand Rest Tremor	34.9	82.5	3/4	87.3	82.5	88.9
Left Leg Rest Tremor	39.4	97.0	1/3	97.0	97.0	97.0
Right Leg Rest Tremor	65.7	97.0	1/2	97.0	97.0	100
Left Hand Postural Tremor	25.4	46.0	3/4	68.3	66.7	76.2
Right Hand Postural Tremor	23.8	73.0	2/4	73.0	74.6	81.0
Left Hand Kinetic Tremor	38.1	60.3	1/3	68.3	71.4	82.5
Right Hand Kinetic Tremor	42.9	77.8	2/3	85.7	85.7	93.7
Left Fingertap (1 target)	43.5	53.2	3/5	54.8	54.8	61.3
Right Fingertap (1 target)	35.5	62.9	2/4	62.9	64.5	64.5
Left Fingertap (2 targets)	29	54.8	3/5	54.8	58.1	61.3
Right Fingertap (2 targets)	35.5	59.7	3/4	62.9	62.9	62.9
Left Pronation/ Supination	33.3	74.6	2/5	74.6	74.6	74.6
Right Pronation/ Supination	39.7	73.0	2/3	77.8	77.8	81.0
Left Leg Agility	20.6	63.5	2/5	65.1	66.7	68.3
Right leg Agility	44.4	69.8	2/4	71.4	79.4	82.5

Overall Mean for All Tests (SEM)	36.7 (4.3)	70.3 (5.9)	-	73.8 (5.3)	74.6 (5.1)	78.7 (5.1)

Supplementary Table 4: LOSO-CV accuracies for the main analysis (any-rater criterion). The accuracy of a number of approaches are compared to a random baseline (similar to rolling a dice where subjects were randomly assigned to a clinical category). The fully prespecified analysis relied on pre-published features and a standard multinomial regression model. The Best Classifier & Prespecified Feature approach selected the best classifier from a range based on best performance but used only the pre-specified features. The Prespecified Classifier & Best Feature approach selected the best feature from a range but used only the pre-specified classifier. The Best Classifier and Feature approach selected the best combination of both. Accuracies are given for each subtest followed by the overall mean (and standard error, SEM). It is also possible for a simple classifier to achieve good performance at the expense of good calibration by predicting a single category consistently. Therefore, for the fully pre-specified analysis the number of categories predicted is shown over the total number of categories in the target sample (i.e. the median clinical score for each subject).

Supplementary Data 2: LOSO-CV Median Prediction analysis (median-rater criterion)

An alternative and more conservative definition of a correct classification is that for an individual prediction, the model was able to agree with the median of the three individual clinical raters (median-rater prediction). Note that a 100% classification accuracy here would mean that the classifier was *better* than any individual rater. Using this criterion, the following LOSO-CV accuracies were obtained which follow a similar pattern to the main analysis but are overall more conservative. Notably the fully prespecified analysis is often worse than the constant baseline on some subtests, but similar overall.

Subtest	Random Baseline	Prespecified Classifier & Feature	Prespecified Classifier & Feature categories predicted	Best Classifier & Prespecified Feature	Prespecified Classifier & Best Feature	Best Classifier & Feature
Left Hand Rest Tremor	34.3	69.9	3/4	66.8	71.4	74.4
Right Hand Rest Tremor	33.3	75.9	3/4	80.6	75.9	82.2
Left Leg Rest Tremor	37.8	95.4	1/3	95.4	95.4	95.4
Right Leg Rest Tremor	62.6	95.5	1/2	95.5	95.5	98.5
Left Hand Postural Tremor	18.7	31.0	3/4	58.3	51.7	71.2
Right Hand Postural Tremor	18.8	71.3	2/4	71.3	72.9	79.3
Left Hand Kinetic Tremor	29.8	53.7	1/3	63.3	58.1	72.5
Right Hand Kinetic Tremor	21.2	52.8	2/3	59.0	59.0	68.7
Left Fingertap (1 target)	24.9	34.6	3/5	37.9	37.9	37.6
Right Fingertap (1 target)	30.4	47.6	2/4	49.3	51.0	49.3
Left Fingertap (2 targets)	17.2	37.9	3/5	37.9	41.1	44.3
Right Fingertap (2 targets)	28.7	44.4	3/4	49.3	49.3	49.3
Left Pronation/ Supination	21.7	41.3	2/5	41.3	41.3	41.3
Right Pronation/ Supination	26.3	56.3	2/3	61.1	57.8	66

Left Leg Agility	15.6	55.2	2/5	56.7	55	56.6
Right leg Agility	34.4	49.8	2/4	51.4	54.4	57.5
Overall Mean for All Tests (SEM)	28.5 (4.7)	57.0 (8.0)	-	60.9 (7.3)	60.5 (7.1)	65.2 (7.5)

Supplementary Table 5: LOSO-CV accuracies for the alternative median prediction analysis. The accuracy of a number of approaches are compared to a random baseline (similar to rolling a dice where subjects were randomly assigned to a clinical category). The fully prespecified analysis relied on pre-published features and a standard multinomial regression model. The Best Classifier & Prespecified Feature approach selected the best classifier from a range based on best performance but used only the pre-specified features. The Prespecified Classifier & Best Feature approach selected the best feature from a range but used only the pre-specified classifier. The Best Classifier and Feature approach selected the best combination of both. Accuracies are given for each subtest followed by the overall mean (and standard error, SEM). It is also possible for a simple classifier to achieve good performance at the expense of good calibration by predicting a single category consistently. Therefore, for the fully pre-specified analysis the number of categories predicted is shown over the total number of categories in the target sample (i.e. the median clinical score for each subject).
